# Longitudinal study of Inflammatory, Behavioral, Clinical and Psychosocial Risk Factors for Chemotherapy-Induced Peripheral Neuropathy

**DOI:** 10.1007/s10549-021-06304-6

**Published:** 2021-06-30

**Authors:** Ian R. Kleckner, Todd A. Jusko, Eva Culakova, Kaitlin Chung, Amber S. Kleckner, Matthew Asare, Julia E. Inglis, Kah Poh Loh, Luke J. Peppone, Jessica Miller, Marianne Melnik, Samer Kasbari, Deborah Ossip, Karen M. Mustian

**Affiliations:** 1. Department of Surgery, Wilmot Cancer Institute, University of Rochester Medical Center, 265 Crittenden Blvd., Box CU 420658, Rochester, NY 14642, USA; 2. Department of Neuroscience, University of Rochester, Rochester, NY, USA; 3. Department of Public Health Sciences, University of Rochester Medical Center, Rochester, NY, USA; 4. Department of Public Health, Baylor University, Waco, TX, USA; 5. Division of Hematology/Oncology, Department of Medicine, Wilmot Cancer Institute, University of Rochester, Rochester, NY, USA; 6. Division of Epidemiology & Community Health, School of Public Health University of Minnesota, Minneapolis, MN, USA; 7. Cancer Research Consortium of West Michigan NCORP, Grand Rapids, MI, USA; 8. Southeast Clinical Oncology Research Consortium (SCOR), Winston Salem, NC, USA

**Keywords:** chemotherapy-induced peripheral neuropathy, CIPN, neuropathy, risk, inflammation

## Abstract

**Purpose.:**

Chemotherapy-induced peripheral neuropathy (CIPN) is a common dose-limiting side effect of taxane and platinum chemotherapy for breast cancer. Clinicians cannot accurately predict CIPN severity partly because its pathophysiology is poorly understood. Although inflammation may play a role in CIPN, there are limited human studies. Here, we identified the strongest predictors of CIPN using variables measured before taxane- or platinum-based chemotherapy, including serum inflammatory markers.

**Methods.:**

116 sedentary women with breast cancer (mean age 55 years) rated (1) numbness and tingling and (2) hot/coldness in hands/feet on 0–10 scales before and after six weeks of taxane- or platinum-based chemotherapy. A sub-study was added to collect cytokine data in the final 55 patients. We examined all linear models to predict CIPN severity at 6 weeks using pre-chemotherapy assessments of inflammatory, behavioral, clinical, and psychosocial factors. The final model was selected via goodness of fit.

**Results.:**

The strongest pre-chemotherapy predictors of numbness and tingling were worse fatigue/anxiety/depression (explaining 27% of variance), older age (9%), and baseline neuropathy (5%). The strongest predictors of hot/coldness in hands/feet were worse baseline neuropathy (11%) and fatigue/anxiety/depression (6%). Inflammation was a risk for CIPN, per more pro-inflammatory IFN-γ (12%) and IL-1β (6%) and less anti-inflammatory IL-10 (6%) predicting numbness/tingling and more IFN-γ (17%) and less IL-10 (9%) predicting hot/coldness in hands/feet.

**Conclusions.:**

The strongest pre-chemotherapy predictors of CIPN included worse fatigue/anxiety/depression and baseline neuropathy. A pro-inflammatory state also predicted CIPN. Because this is an exploratory study, these results suggest specific outcomes (e.g., IL-1β) and effect size estimates for designing replication and extension studies.

**Trial Registration**. NCT00924651

## Background.

Over half of all patients with breast cancer receiving taxane- and platinum-based chemotherapy regimens experience chemotherapy-induced peripheral neuropathy (CIPN) [1, 2]. CIPN affects the hands and feet with sensory symptoms including numbness, tingling, and cold-induced, burning, and sharp-shooting pain; motor symptoms including cramping, difficulty handling small objects, and balance issues; and autonomic symptoms including dizziness [2, 3]. CIPN is a dose-limiting toxicity, thereby potentially increasing mortality [4], and it interferes with activities of daily living and reduces quality of life. CIPN symptoms typically escalate over the weeks of chemotherapy [5] and recover slowly. Nearly half of all CIPN patients have symptoms six months after completing chemotherapy [2], and many require years to recover, if they recover at all [6]. Two key challenges in CIPN research are (1) clinicians cannot accurately predict which patients will experience the worst CIPN before initiating chemotherapy, and (2) there are very limited treatments for CIPN despite nearly 100 clinical trials in humans [7, 8], likely due to its poorly understood pathophysiology [9]. One way to help address these challenges is by conducting epidemiological studies to suggest (1) risk factors for CIPN and (2) mechanisms potentially related to the pathophysiology of CIPN, which can inform new interventions.

Previously implicated risk factors for CIPN that have been consistently observed across studies include pre-existing or early-onset symptoms of neuropathy (e.g., [10–14]), anxiety or depression (e.g., [13, 15]), and sedentary behavior (e.g., [16–18]); for a review of other CIPN risk factors, see [2, 14]. Some risk factors inconsistently observed across studies include older age (e.g., null findings in [10–12, 19]; positive findings in [16, 20]; opposite trend in [21]) and diabetes status (e.g., null findings in [12, 22, 23]; positive findings in [24]).

Although the pathophysiology of CIPN likely involves many factors [2, 9, 25], a growing body of evidence suggests that inflammation plays an important role [26–30]. Experimental studies in non-human animals have provided most of this evidence. For example, studies in mice show that pro-inflammatory cytokines (e.g., IL-1β, tumor necrosis factor-α [TNF-α]) damage [31] affect the excitability [32] of peripheral nerves and thus lead to CIPN symptoms, such as hyperalgesia [32]. Another study in mice found that paclitaxel up-regulates inflammatory cytokines (IFN-γ, TNF-α, and others) in the spine concurrent with symptoms of CIPN [33].

Human research on inflammation and CIPN has been limited to a few case-control studies. A small study in 17 patients with breast cancer suggested that reduced IL-6 before chemotherapy is associated with greater symptoms of CIPN after chemotherapy [34]. A study in 190 patients of mixed cancer types found that CIPN was more severe when anti-inflammatory drugs were withheld during infusion [35]. And finally, a study of 67 patients with breast cancer found that those with CIPN had markers of systemic inflammation (e.g., higher neutrophil-to-lymphocyte-ratio) compared to patients without CIPN [36]. Given that these studies are cross-sectional or small, additional studies are needed to investigate the relationship between CIPN and inflammation in humans.

The goals of this secondary analysis [37] are (1) to examine potential risk factors of developing CIPN using variables measured before chemotherapy, hopefully to assist clinicians in identifying who will experience CIPN before it occurs, and (2) to explore whether inflammation plays a role in CIPN in humans. We assessed patient-reported neuropathy symptoms before and after 6 weeks of neurotoxic taxane- or platinum-and taxane based chemotherapy in 116 female patients with breast cancer. The primary outcome for this study was numbness and tingling, and the secondary outcome was hot/coldness in the hands and feet. Potential risk factors were assessed before chemotherapy and include inflammation (serum cytokine concentration), clinical factors (chemotherapy type, cancer stage, body mass index [BMI], fatigue, anxiety, depression, use of diabetes medications), behavioral factors (physical activity), and demographic factors (age, race). We did not use a full CIPN questionnaire or assess pain because the parent study did not include a CIPN questionnaire or assessments of CIPN-specific pain (e.g., burning/shooting pain in the hands or feet). We hypothesized that more severe CIPN after 6 weeks of neurotoxic chemotherapy would be predicted by baseline (pre-chemotherapy) pro-inflammatory state (elevated IL-6, decreased IL-10), older age, worse fatigue, worse anxiety, worse depression, use of diabetes medications, and lower physical activity levels. This is an exploratory analysis that can help generate hypotheses, and inform outcomes and provide effect sizes for sample size calculations in future studies [37].

## Materials and Methods

### Study design

This was a secondary analysis [37] of patients with breast cancer randomized to usual care from a randomized clinical trial designed to assess the effects of exercise on fatigue among patients with cancer receiving chemotherapy (ClinicalTrials.gov NCT00924651; for details see [38–41]). The trial was conducted through the University of Rochester Cancer Center (URCC) National Cancer Institute (NCI) Community Oncology Research Program (NCORP) Research Base across 20 community oncology practices in the United States from 2009–2014. URCC and all community oncology institutional review boards approved the study before participants were enrolled. All participants provided written informed consent before beginning study assessments. All baseline measures were completed the week before randomization (week 0) and all final measures were obtained during week 6.

### Study participants

To be eligible for the parent study, patients must have (1) been ≥21 years old, (2) had a primary diagnosis of cancer other than leukemia, without distant metastasis, (3) been chemotherapy naïve, (4) started chemotherapy after enrollment and been scheduled for at least 6 weeks of chemotherapy with treatment cycles of either 2, 3, or 4 weeks long; (5) had a Karnofsky Performance Status ≥70, (6) been able to read English, (7) not received concurrent radiation therapy, (8) not had physical limitations that contraindicate participation in a low- to moderate-intensity home-based walking and progressive resistance program (determined by the patient’s oncologist, who had full knowledge of the program), and (9) not been identified as in the active or maintenance stage of exercise behavior as assessed by the Exercise Stages of Change [42]. This exploratory secondary analysis included all 116 patients with breast cancer randomized to usual care, received taxane- and/or platinum-based chemotherapy, and rated their neuropathy symptoms before and after 6 weeks of chemotherapy. We excluded patients randomized to exercise because this exercise intervention influenced multiple markers of inflammation (i.e., our assessed risk factors) [41].

### Measures

Neuropathy symptoms were assessed via patient-reported numbness and tingling (primary outcome) and hot/coldness in hands/feet (secondary outcome) using 0–10 scales, where 0 = “symptom not present” and 10 = “symptom as bad as you can imagine” during the last seven days. Validity and reliability have been demonstrated for similar scales [15, 43]. The parent study did not include a full CIPN questionnaire or assessments of CIPN-specific pain. Patients were instructed to fast for morning blood draws and we used a Luminex MagPix (Austin, TX) to analyze concentrations of serum cytokines IL-1β, IL-6, IL-8, IL-10, and IFN-γ, and receptor sTNFR1; for more detail see [41]. Measures of demographic and clinical factors were abstracted from medical records (chemotherapy type, cancer stage, and BMI) and patient report (age, race). We assessed fatigue using the multidimensional fatigue symptom inventory (MFSI) short form, which is a valid, reliable 30-item measure [44]. We assessed depression using the Center for Epidemiological Studies Depression Scale (CES-D) [45], a 20-item scale shown to reliably and validly measure depression in cancer populations [46]. We assessed anxiety using the Spielberger State/Trait Anxiety Inventory (STAI Form Y-1; i.e., only *state* anxiety) [47], a 20-item anxiety scale used in patients with cancer [46, 48]. Physical activity was measured using a pedometer (Walk 4 Life Classic; Oswego, IL) worn on the waist for all waking hours of four days before the first chemotherapy infusion.

#### Cytokine data processing.

All cytokine data were log_10_ transformed to reduce skew. We then removed 4 outliers that were greater than 3 standard deviations from the mean: 1 from log_10_(sTNFR1), 1 from log_10_(IL-8), and 2 from log_10_(IL-10). Each cytokine had its own detection limit based on the measurement methodology. Nearly half the data were below the detection limit for IL-1β and IFN-γ so they were set to categorical variables as described below.

#### Symptom cluster of fatigue, anxiety, and depression.

**Figure 1.**
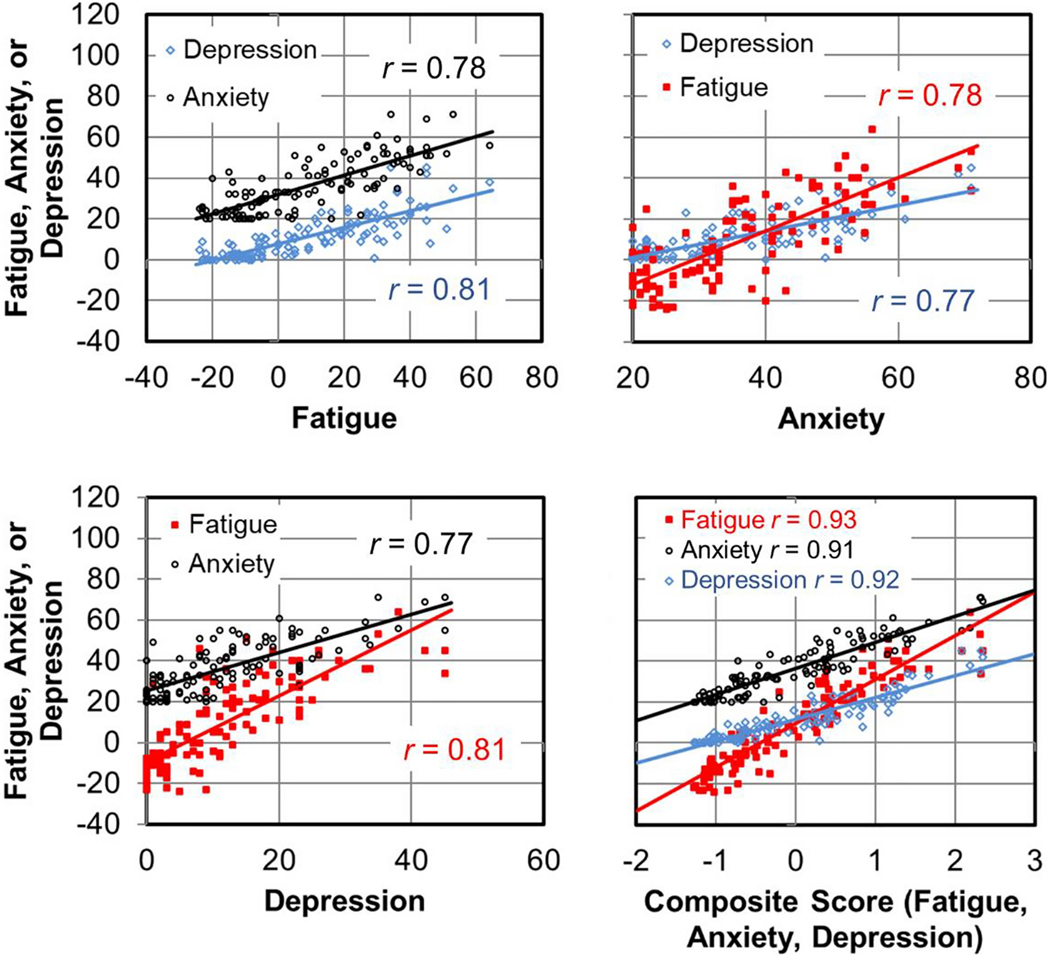
Patient-reported fatigue (MFSI), anxiety (STAI), and depression (CES-D) were all highly related via Person’s correlation and thus were standardized and averaged into a single composite score. Each data point is from one of the 116 patients before starting neurotoxic chemotherapy.

In our bivariate analyses of potential risk factors, we observed a clustering of patient-reported fatigue, anxiety, and depression. Thus, we standardized each variable as a z-score and averaged the three standardized variables into a single composite score (Figure 1).

#### Statistical analyses.

Analyses were performed using JMP software v.13 (SAS Institute Inc.; Cary, NC). Due to the exploratory nature of this work, we did not adjust for multiple comparisons to prevent Type II errors [49], i.e., incorrectly concluding that a true risk factor for CIPN is not associated with CIPN. To examine changes in CIPN, we used paired t-tests. To examine risk factors for CIPN, we used multiple linear regression to model post-intervention CIPN symptoms—(a) numbness and tingling or (b) hot/coldness in hands/feet—using an intercept term and all possible non-interaction combinations of risk factors. Our results describe the model with the lowest AICc (designated the final model) to balance competing goals of increasing goodness of fit and reducing the number of parameters [50]. In total, the potential risk factors were: *baseline neuropathy symptoms* (continuous; (a) numbness and tingling or (b) hot/coldness in hands/feet, to match the outcome), *inflammation (where each cytokine had its own detection limit based on measurement methodology)*: serum concentration of IL-1β (categorical: not detected vs. low vs. high where low and high were determined via median split on detected values), IL-6 (continuous), IL-8 (continuous), IL-10 (continuous), IFN-γ (detected vs. not detected), and sTNFR1 (continuous), *clinical factors*: neurotoxic chemotherapy type (categorical: only taxane vs. taxane and platinum), cancer stage (categorical: I vs. II, III, and IV), BMI (continuous), receiving diabetes medications (categorical: yes vs. no), composite score of z scores of fatigue, anxiety, and depression (continuous); *behavioral factors*: pedometer steps per day (continuous), and *demographics*: age (continuous), race (categorical: black vs. non-black). We reported the two final models (one for each CIPN outcome).

We also report our analyses on two separate sub-cohorts: all patients (*N* = 116), and only patients who provided a blood specimen (*N* = 55). Blood collection only became available halfway through enrollment, and 89% of patients approached agreed to provide blood.

## Results

### Baseline characteristics (Table 1)

The 116 women with breast cancer were on average middle-aged (55 years), obese (BMI 30.3 kg/m^2^), sedentary (4,412 steps/day), white (82%), employed outside the house (55%), married or in a committed relationship (66%), and had at least some college education (67%). Most cancer was early-to-mid stage (I, II, and III comprised 35%, 53%, and 10%, respectively) with high Karnofsky Performance Status (59% scored 100). Nearly all patients (91%) had previously received surgery for their cancer, on average 6 weeks before starting chemotherapy. All patients received neurotoxic chemotherapy with the majority being only taxane-based (70%), followed by combined taxane- and platinum-based (30%).

### Severity and prevalence of neuropathy symptoms

Before chemotherapy, the average severity of numbness and tingling was 1.14 and the average severity of hot/coldness in hands/feet was 0.92 on a 0–10 point scale (Table 2). After six weeks of neurotoxic chemotherapy, CIPN symptom severity significantly increased by 0.64 points (95% CI: 0.17, 1.11) for numbness and tingling and by 0.72 points for hot/coldness in hands/feet (95% CI: 0.29, 1.14). When dichotomizing CIPN symptoms as absent or present (per Bao et al., 2016; [15]), we observed an increase of 17% in prevalence of numbness and tingling from 36% to 53%. For hot/coldness in hands/feet, we observed an increase of 16% in prevalence from 27% to 43%. The same trends in severity and prevalence over time held in both sub-cohorts (Table 3).

### Assessment of potential risk factors excluding inflammation

**Figure 2.**
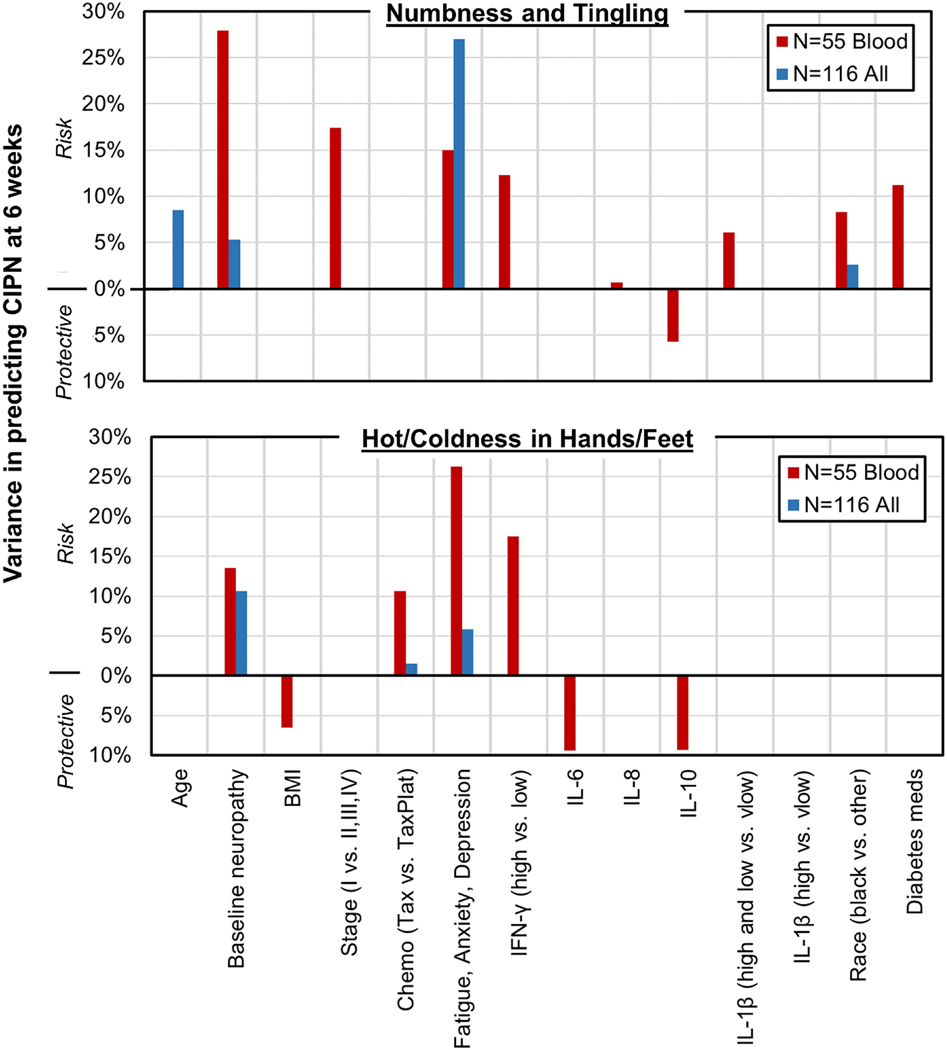
Variance explained for each potential risk factor in explaining numbness/tingling (top) or hot/coldness in hands/feet (bottom) for the two subcohorts. Risk factors are shown with percentage variance pointing upwards. Protective factors are shown with percentage variance pointing downwards.

First, we examined the full cohort of 116 patients without the requirement for blood specimens to assess inflammation. The final model for numbness and tingling included worse fatigue/anxiety/depression (which explained 27% of variance in CIPN severity), older age (9%), worse baseline neuropathy (5%), and black race (3%). ^1^The final model for hot/coldness in hands/feet included worse baseline neuropathy (11% of variance), fatigue/anxiety/depression (6%), and taxane chemotherapy (2%). The model selection procedures consistently rejected several variables as potential risk factors for CIPN symptom severity: cancer stage, BMI, use of diabetes medications, and pedometer steps (Figure 2).

### Assessment of potential risk factors including inflammation

In all 55 patients who provided a blood specimen (Table 5), the final model for numbness/tingling included worse baseline neuropathy (28%), stage I cancer (17%), worse fatigue/anxiety/depression (15%), higher pro-inflammatory IFN-γ (12%), the use of diabetes medications (11%), black race (8%), higher pro-inflammatory IL-1β (6%), lower anti-inflammatory IL-10 (6%) and higher pro-inflammatory IL-8 (1%). The final model for hot/coldness in hands/feet included worse fatigue/anxiety/depression (26%), higher pro-inflammatory IFN-γ (18%), worse baseline neuropathy (14%), taxane chemotherapy (11%), lower IL-6 (9%), lower anti-inflammatory IL-10 (9%), and lower BMI (6.5%).

Taken together, these data suggest several pro-inflammatory markers that were significant risk factors for CIPN (IFN-γ, IL-1β, IL-8) and an anti-inflammatory marker that was protective against CIPN (IL-10), and IL-6, which was protective and has both pro- and anti-inflammatory properties [51]. These predictors each explained 1–18% of variance in the context of the other predictors in the final models. The potential risk factors for CIPN that were consistently rejected in our model selection procedure included sTNFR1, pedometer steps, and age (Figure 2).

## Discussion

We explored several risk factors for predicting CIPN symptom severity after 6 weeks of neurotoxic taxane or platinum chemotherapy in women with breast cancer by assessing pre-chemotherapy inflammatory, behavioral, clinical and psychosocial risk factors. We found several consistent risk factors for CIPN including more severe baseline neuropathy, more severe fatigue/anxiety/depression, higher levels of pro-inflammatory IFN-γ, and lower levels of anti-inflammatory IL-10, and black race.

This is the largest and among the first study in human patients with cancer (not preclinical models, e.g. [33]) suggesting that a pro-inflammatory state before chemotherapy is a risk factor for CIPN. We found that higher levels of pro-inflammatory IFN-γ and lower levels of anti-inflammatory IL-10 in serum were both risk factors for more severe CIPN, based on both patient-reports for the primary outcome numbness and tingling and the secondary outcome hot/coldness in hands/feet. Other risk factors included higher pro-inflammatory IL-1β (for numbness and tingling), pro-inflammatory IL-8 (for numbness/tingling), and lower IL-6 (for hot/coldness), which has both pro- and anti-inflammatory properties [51]. Our results are consistent with several prior studies: (1) one suggesting that blocking IL-1β signaling reduced CIPN symptoms in rats who received paclitaxel [52]; (2) a correlational study in humans suggesting that a single IL-1β polymorphism is a risk factor for CIPN [53]; and (3) a case-control study in humans suggesting perturbed gene expression in neuroinflammatory pathways in breast cancer survivors with CIPN vs. those without CIPN [54].

These cytokines might contribute to CIPN via known effects of inflammatory cytokines of excitability of peripheral nerves [31, 32]. Specifically, IFN-γ causes neuronal hypersensitivity by activating microglia and has been implicated in CIPN symptoms such as neuropathic pain [55]. Next, IL-1β affects GABA receptor activity in the brain [56], which is consistent with evidence that inflammation and reduced GABA levels in the brain contribute to CIPN symptoms [57]. Finally, IL-10 not only downregulates production of proinflammatory cytokines [58] but it also down-regulates sodium channel activity in the dorsal root ganglia [59], which is implicated in the etiology of CIPN [60]. Taken together, our observations lend support and add specificity to theories that inflammation is involved in the etiology of CIPN in humans [26–28, 61], and can help advance and inform anti-inflammatory treatments for CIPN [62, 63].

Our results suggest that one of the strongest risk factors for CIPN symptom severity is the symptom cluster of fatigue, anxiety, and depression (typically accounting for 18% variance), consistent with prior research [13, 15]. These results invite the hypothesis that there is a shared biological mechanism of fatigue, anxiety, depression, and CIPN; the mechanism might be peripheral inflammation, neuroinflammation via disrupted blood-brain-barrier integrity [64], changes in the brain [65–67] including the interoceptive brain system, which processes bodily sensations [68], or mitochondrial dysfunction [69], all of which are implicated in the etiology of not only CIPN (for discussion, see [38]) but also fatigue, anxiety, and depression [69–71]. Furthermore, if fatigue, anxiety, and depression play causal roles in the development of CIPN symptoms, then interventions to reduce fatigue, anxiety, and depression could help prevent or alleviate symptoms of CIPN [72]. Potential interventions include (1) exercise, which improves depression [73], anxiety [73], fatigue [74], inflammation [75], and affects the interoceptive brain system (e.g., [76]), and may reduce CIPN [38, 39] (for a review, see [72, 77–79]); (2) pharmaceuticals such as duloxetine, which improves depression [80] and CIPN [81]; (3) neuromodulation or neurofeedback, which improves depression [82–84] and may also improve CIPN (e.g., [85]).

Our weakest observed risk factors for CIPN include age and the use of diabetes medications, consistent with mixed findings in the literature (see Introduction for citations). The mixed findings may be due to statistically weak associations and across-study differences in samples, sample sizes, use of a cutoff vs. continuous measures for age, and choice of other covariates. Some of the risk factors for CIPN that we observed might be due to confounding chemotherapy factors such as type and dose, which we did not collect. Physical activity (pedometer steps) and BMI were both weak or non-significant predictors of CIPN severity despite prior studies indicating that physical activity is protective [16–18] and BMI is a risk factor [16, 20]. Our unexpected observations may be because our eligibility criteria ensured that our sample was sedentary (low steps, high BMI), thus restricting variability in physical activity levels. Our observation that black patients were at greater risk for CIPN severity was consistent with prior work [16], and is perhaps due to undiagnosed or untreated comorbidities such as prediabetes or diabetes, which is more prevalent in black Americans compared to white Americans [86–88].

This study has several strengths. First, to our knowledge, this is the largest longitudinal human study of inflammation and CIPN (116 patients, with 55 patients providing blood for inflammation measures; the only prior study involved 17 patients [34]). Second, assessing CIPN before and after six weeks of chemotherapy provides stronger evidence for CIPN risk factors than a cross-sectional post-chemotherapy design. Third, our data were obtained from community oncology clinics around the United States, enhancing the generalizability to our findings compared to single-site studies.

This study also has limitations. First, because this was an exploratory secondary analysis [37] based on subpopulation of 116 patients randomized to usual care from a randomized clinical trial, these findings should be tested for replication and further validation. With that said, our pattern of findings with pro- and anti-inflammatory cytokines are as expected based on prior literature and presumed mechanisms, lending credibility to these results. Second, the measures of CIPN were crude, with two 0–10 patient-reported questions instead of a full multi-question survey designed to assess CIPN. However, the measures we used have been successfully used and validated in prior studies (e.g., [15, 43]). Moreover, we did not have access to clinical assessment of CIPN (e.g., monofilament test, nerve conduction) specific to CIPN. Third, we only measured the first 6 weeks of chemotherapy and therefore the severity of neuropathy in our sample was relatively low. However, our 6-week measure may serve as a surrogate for future CIPN severity, as paclitaxel causes a monotonic weekly increase in CIPN severity (e.g., Figure 4 from [89]). Fifth, our precision may be reduced given small cell sizes in some variables (e.g., only a few black patients). In addition, we do not have information on certain variables that might be related to neuropathy symptoms, such as smoking status, alcohol consumption, HIV status, and chemotherapy dose. Sixth, our assessment of serum cytokines may not necessarily reflect inflammation in the nervous system that contributes to CIPN. However, serum inflammation is convenient to assess and could serve as a useful biomarker of CIPN in the future. In addition, detection limits of cytokine assessment methodology forced us to use categorical coding for some cytokines (e.g., IL-1β, IFN-γ). However, we still saw prediction of CIPN severity with cytokines that were not subject to categorical coding (e.g., IL-10, IL-6). Finally, it is not known how these results generalize to other populations, as our sample was primarily white, well-educated, female patients with breast cancer who were sedentary and reported mild (not severe) neuropathy.

## Conclusion

In this exploratory secondary analysis involving 116 patients, the strongest risk factors for development of CIPN after 6 weeks of neurotoxic taxane or platinum chemotherapy included more severe baseline neuropathy and the symptom cluster of fatigue, anxiety, and depression. Our data in 55 patients also suggest that a pro-inflammatory state before chemotherapy is a significant risk factor for CIPN, making this the largest and among the first longitudinal studies of inflammation and CIPN in humans. Our findings invite the hypothesis of a common biological mechanism (e.g., inflammation, brain connectivity, mitochondrial dysfunction) that underlies the etiology of CIPN, as well as fatigue, anxiety, and depression. Given the exploratory nature of this work, future studies should test for replication. Ultimately, identifying the strongest predictors of CIPN can help clinicians inform patients of the risks of chemotherapy as well as identify and optimize promising mechanism-based treatments for CIPN.

## Figures and Tables

**Table 1.** Patient baseline characteristics.

Sample Characteristic	All	Patients who provided blood
***Total participants***	116	55
***Age, years (M ± SD)***	55.4 ± 9.8	56.7 ± 9.1
***Body mass index, kg/m^2^ (M ± SD)***	30.3 ± 6.1	29.7 ± 6.1
***Daily steps from pedometer (M ± SD)***	4,412 ± 2961	4,695 ± 3,192
***Time since end of first radiation or surgery for cancer, weeks (M ± SD)***	6.4 ± 12.4	4.9 ± 7.0
***Race***		
*White*	95 (82%)	46 (84%)
*Black*	14 (12%)	7 (13%)
*Other*	7 (6%)	2 (4%)
***Employment***		
*Employed outside the house*	64 (55%)	35 (64%)
*Unemployed*	39 (34%)	15 (27%)
*Self-employed / homemaker*	13 (11%)	5 (9%)
***Marital status***		
*Married or long-term committed relationship*	76 (66%)	31 (56%)
*Divorced, separated, single, widowed*	40 (34%)	24 (44%)
***Education***		
*At least some college*	78 (67%)	34 (62%)
*High school/GED degree*	33 (28%)	18 (33%)
*No high school or GED degree*	4 (3%)	2 (4%)
*Unknown*	1 (1%)	1 (2%)
***Cancer stage***		
*Stage I*	41 (35%)	20 (36%)
*Stage II*	62 (53%)	31 (56%)
*Stage III*	12 (10%)	
*Stage IV*	0 (0%)	
*Unknown*	1 (1%)	0 (0%)
***Previous treatment***		
*Previous surgery*	105 (91%)	50 (91%)
*Previous hormone therapy*	2 (2%)	2 (4%)
*Previous radiation therapy*	2 (2%)	1 (2%)
***Neurotoxic chemotherapy type***		
*Taxane-based only*	81 (70%)	42 (76%)
*Platinum- and taxane-based*	35 (30%)	13 (24%)
***Karnofsky Performance Status***		
*80*	10 (9%)	7 (13%)
*90*	37 (32%)	18 (33%)
*100*	69 (59%)	30 (55%)

**Table 2.** Patient-reported severity of neuropathy symptoms before and after 6 weeks of neurotoxic chemotherapy.

	Before chemotherapy		After 6 weeks of chemotherapy		Change (after minus before)		

	Mean	95% CI	Mean	95% CI	Mean	95% CI	p-value
***Sample: all 116 patients***							
*Severity of numbness and tingling in the past week (0– 10)*	1.14	0.76, 1.52	1.78	1.34, 2.21	0.64	0.17, 1.11	0.0089
*Severity of hot/coldness in hand/feet in the past week (0–10)*	0.92	0.56, 1.29	1.64	1.16, 2.11	0.72	0.29, 1.14	0.0013
***Sample: all 55 patients who provided blood***							
*Severity of numbness and tingling in the past week (0–10)*	0.98	0.48, 1.48	1.71	1.07, 2.35	0.73	0.18, 1.28	0.0124
*Severity of hot/coldness in hand/feet in the past week (0–10)*	1.07	0.47, 1.67	1.84	1.08, 2.59	0.76	0.17, 1.35	0.0139

**Table 3.** Patient-reported prevalence of neuropathy symptoms before and after 6 weeks of neurotoxic chemotherapy.

	Before chemotherapy	After 6 weeks of chemotherapy	Change (after minus before)
	
***Sample: all 116 patients***			
*Prevalence of numbness and tingling in the past week (1–10)*	36%	53%	17%
*Prevalence of hot/coldness in hand/feet in the past week (1–10)*	27%	43%	16%
***Sample: all 55 patients who provided blood***			
*Prevalence of numbness and tingling in the past week (1–10)*	36%	51%	15%
*Prevalence of hot/coldness in hand/feet in the past week (1–10)*	27%	42%	15%

**Table 4.** Model fit using stepwise regression for outcome of patient-reported severity of neuropathy symptoms in all 116 patients before and after 6 weeks of neurotoxic chemotherapy.

Term ^a^	Estimate ^b^	95% CI	p-value	η_p_^2^
***Outcome: Severity of numbness and tingling in the past week (0–10)***				
*Intercept*	−1.98	−4.11, 0.15	0.068	-
*Composite of Fatigue, Anxiety, Depression*	1.32	0.91, 1.72	<.0001	27.0%
*Age (years)*	0.06	0.02, 0.10	0.002	8.5%
*Baseline neuropathy (numbness & tingling)*	0.23	0.05, 0.41	0.014	5.3%
*Race (black minus other)*	0.97	−0.14, 2.08	0.085	2.6%
***Outcome: Severity of hot/coldness in hands/feet in the past week (0–10)***				
*Intercept*	0.78	0.01, 1.55	0.048	-
*Baseline neuropathy (hot/coldness)*	0.47	0.21, 0.73	0.0004	10.6%
*Composite of Fatigue, Anxiety, Depression*	0.71	0.17, 1.24	0.010	5.8%
*Chemotherapy type (Taxane minus Taxane+Platinum)*	0.58	−0.29, 1.46	0.190	1.5%

a Predictor variables arranged from those most strongly predictive of the outcome (top) to those less strongly predictive of the outcome (bottom) based on partial eta squared, η_p_^2^.

b Positive coefficients indicate more severe CIPN symptoms for the reference group or increasing values of the predictor variable.

**Table 5.** Model fit using stepwise regression for outcome of patient-reported severity of neuropathy symptoms in all 55 patients who provided a blood sample before and after 6 weeks of neurotoxic chemotherapy.

Term ^a^	Estimate ^b^	95% CI	p-value	η_p_^2^
***Outcome: Severity of numbness and tingling in the past week (0–10)***				
*Intercept*	4.35	1.01, 7.68	0.012	-
*Baseline neuropathy (numbness & tingling)*	0.55	0.28, 0.81	0.0001	27.9%
*Cancer stage (1 minus 2, 3, and 4)*	1.48	0.51, 2.44	0.004	17.4%
*Composite of Fatigue, Anxiety, Depression*	0.65	0.18, 1.11	0.007	15.0%
*IFN-γ (high minus low)*	1.16	0.23, 2.09	0.016	12.3%
*Using diabetes medications*	1.97	0.30, 3.64	0.022	11.2%
*Race (black minus other)*	1.40	0.00, 2.79	0.050	8.3%
*IL-1β (high and low minus very low)*	0.42	−0.07, 0.92	0.093	6.1%
*IL-10*	−1.33	−2.96, 0.29	0.105	5.7%
*IL-8*	0.44	−1.13, 2.02	0.573	0.7%
***Outcome: Severity of hot/coldness in hands/feet in the past week (0–10)***				
*Intercept*	6.46	2.65, 10.26	0.001	-
*Composite of Fatigue, Anxiety, Depression*	1.37	0.66, 2.09	0.0004	26.3%
*IFN-γ (high minus low)*	1.74	0.56, 2.91	0.005	17.5%
*Baseline neuropathy (hot/coldness)*	0.46	0.10, 0.82	0.014	13.5%
*Chemotherapy type (Taxane minus Taxane + Platinum)*	1.48	0.15, 2.82	0.031	10.6%
*IL-6*	−1.66	−3.27, −0.06	0.043	9.4%
*IL-10*	−1.89	−3.72, −0.05	0.044	9.3%
*BMI*	−0.08	−0.16, 0.01	0.095	6.5%

a Predictor variables arranged from those most strongly predictive of the outcome (top) to those less strongly predictive of the outcome (bottom) based on partial eta squared, η_p_^2^.

b Positive coefficients indicate more severe CIPN symptoms for the reference group or increasing values of the predictor variable.

## Footnotes

1 The percent variance refers to predictive ability of predictor variable (not the percent of increase in risk for CIPN, which could be inferred using a different model or interpreting the extent of change in CIPN severity for a given amount of change in the Estimate parameter in the tables.
